# Gene expression profiling reveals upregulated *FUT1* and *MYBPC1* in children with pancreaticobiliary maljunction

**DOI:** 10.1590/1414-431X20198522

**Published:** 2019-07-29

**Authors:** Wan-Liang Guo, Jia Geng, Jun-gang Zhao, Fang Fang, Shun-Gen Huang, Jian Wang

**Affiliations:** 1 Department of Radiology, Children's Hospital of Soochow University, Suzhou, China; 2 Clinical Laboratory, the 3rd Hospital of Yulin, Yulin, China; 3 Department of Pediatric Surgery, Children's Hospital of Soochow University, Suzhou, China

**Keywords:** Pediatric, Pancreaticobiliary maljunction, Gene expression

## Abstract

Pancreaticobiliary maljunction (PBM) is associated with high risk of epithelial atypical growth and malignant transformation of the bile duct or gallbladder. However, overall changes in genetic expression have not been examined in children with PBM. Genome-wide expression was analyzed using peripheral blood samples from 10 children with PBM and 15 pediatric controls. Differentially expressed genes (DEGs) were identified using microarray. Bioinformatics analysis was conducted using Gene Ontology and KEGG analyses. The top 5 in the up-regulated genes in PBM were verified with qRT-PCR. Receiver operator characteristic curve analysis was conducted to evaluate the predictive accuracy of selected genes for PBM. The microarray experiments identified a total of 876 DEGs in PBM, among which 530 were up-regulated and the remaining 346 were down-regulated. Verification of the top 5 up-regulated genes (*TYMS*, *MYBPC1*, *FUT1*, *XAGE2*, and *GREB1L*) by qRT-PCR confirmed the up-regulation of *MYBPC1* and *FUT1*. Receiver operating characteristic curve analysis suggested that *FUT1* and *MYBPC1* up-regulation could be used to predict PBM, with the area under the curve of 0.873 (95%CI=0.735−1.000) and 0.960 (95%CI=0.891−1.000), respectively. *FUT1* and *MYBPC1* were up-regulated in children with PBM, and could be used as potential biomarkers for PBM.

## Introduction

Pancreaticobiliary maljunction (PBM) is a congenital anomaly characterized by the junction of the pancreatic and biliary ducts outside the duodenal wall (1,2). Due to the lack of control of pancreaticobiliary junction by the sphincter of Oddi, reciprocal reflux of bile and pancreatic juice occurs. Reflux of pancreatic juice into the bile duct damages the bile duct wall and could lead to bile duct dilatation (3,4). PBM is often accompanied by pancreatitis, cholestasis, stone formation, epithelial hyperplasia, and malignant transformation in the biliary tract (5–7). Once a diagnosis of PBM is established, prophylactic surgery (mostly in the form of cyst excision and Roux-en-Y hepaticojejunostomy) is recommended to prevent carcinogenesis (8,9).

In a large case series report by Tashiro et al. (10), 11% of the PBM patients had cancer in the biliary system, 2/3 in the gallbladder and the remaining 1/3 in the bile duct. Such a trend is more pronounced in PBM without choledochal cysts. Kaneko et al. (11) reported association of certain genes with the development of gallbladder cancer in PBM. In the current study, we examined whether gene expression profile could be used for early detection of PBM itself. Specifically, we conducted genome-wide transcriptional profiling using blood-derived mRNA followed by real-time PCR verification in 10 children with PBM versus 15 control cases.

## Material and Methods

### Study subjects

The study protocol was approved by the Institutional Review Board of the Children's Hospital of Soochow University (No. 20160106011), in compliance with the Declaration of Helsinki. There was no ethical/legal conflict involved in the study. Written informed consent was obtained from the legal guardians of all subjects.

### RNA extraction

Peripheral blood mononuclear cells were isolated, and then stored at –80°C before RNA extraction using TRIZOL (Invitrogen, USA).

### Microarray analysis

Microarray experiments were conducted by Shanghai KangChen Biotech (China; http: //http:/www.kangchen.com.cn) with Agilent Human 4x44K Gene Expression Microarray chips with >44,000 probes using the Agilent One-Color Microarray-Based Gene Expression Analysis protocol.

### Real-time reverse transcription-polymerase chain reaction assay

Real-time quantitative PCR was used to determine the level of mRNAs for the *TYMS*, *MYBPC1*, *FUT1*, *XAGE2*, and *GREB1L* genes using a SYBR Green PCR Kit (Applied Biosystems, USA). The sequence of primers is listed in Table 1. The relative expression of gene transcript was calculated using the 2^−ΔΔCt^ method, and was normalized to β-actin mRNA.

**Table 1.** Upstream and downstream primer sequences.

Gene name	Primer sequences	Annealing temperature (°C)	Product length (bp)
*β-actin* (H)	F: 5′ GTGGCCGAGGACTTTGATTG 3′R: 5′ CCTGTAACAACGCATCTCATATT 3′	60	73
*MYBPC1*	F: 5′ GACTGGACCCTTGTCGAAACT 3′R: 5′ TCTTCACCAACTTTCACTGTTCC 3′	60	119
*FUT1*	F: 5′ GACATTGGCTAAGCCTTGA 3′R: 5′ AAGATCAGGCTACTTCAGAAAG 3′	60	53
*GREB1L*	F: 5′ GGACAAGCGATTTCTACCA 3′R: 5′ TCTTTCTCCACAGCCGATA 3′	60	82
*XAGE2*	F: 5′ TAGGCCAAGAAGAAGTTTACAG 3′R: 5′ CATCAGTGGGTTCAAGCATAG 3′	60	62
*TYMS*	F: 5′ GTGCATTTCAATCCCACG 3′R: 5′ GACGAATGCAGAACACTTCT 3′	60	216

H: human.

### Statistical analysis

SPSS 20.0 software (IBM, USA) was used for statistical analysis. Continuous variables are reported as means±SD, and were analyzed using the rank sum test. Receiver operator characteristic (ROC) curve analysis was conducted and area under the ROC curve (AUC) was used to evaluate the predictive accuracy of selected genes for PBM. P<0.05 was considered statistically significant.

## Results

### Demographic and clinical data

A total of 10 pediatric PBM cases (age: 65.2±36.6 months) and 15 controls (age: 74.1±38.8 months) were included in the study. Demographic and clinical data are summarized in Table 2.

**Table 2.** Demographic and clinical characteristics of pediatric pancreaticobiliary maljunction.

Variables	Todani types I (n=7)	Todani types IV (n=3)
Abdominal pain	6	3
Jaundice	4	1
Mass	1	0
Fever	2	1
Vomiting	4	1
Gender (male)	2	0
Age (months)	22−105	60−85
Pathological findings		
Cyst wall hyperplasia	7	3
Gallbladder wall congestion	7	3

### Differential gene expression

**Figure 1.**
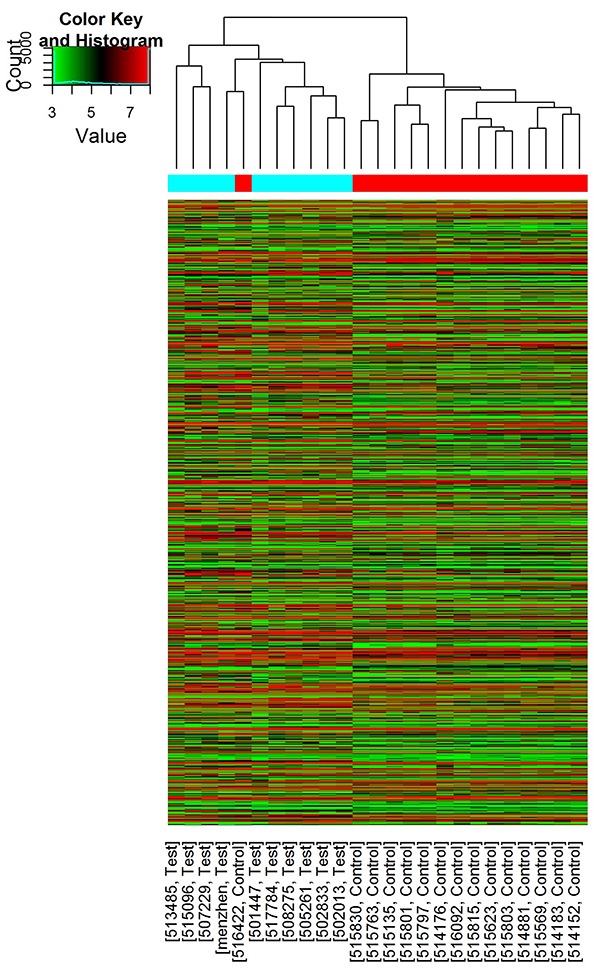
Hierarchical clustering showing mRNA expression profile between the two groups and homogeneity within each group (blue: pancreaticobiliary maljunction (PBM); red: control). Red and green represent up-regulated and down-regulated genes in the PBM group.

The microarray experiment identified a total of 876 differentially expressed genes (DEGs; cutoff at 2-fold): 530 were up-regulated and the remaining 346 were down-regulated in subjects with PBM. The heat maps showed distinct gene expression profile in PBM compared with the normal control (Figure 1). Homogeneity within the 2 groups was fair.

### GO and pathway analysis

**Figure 2.**
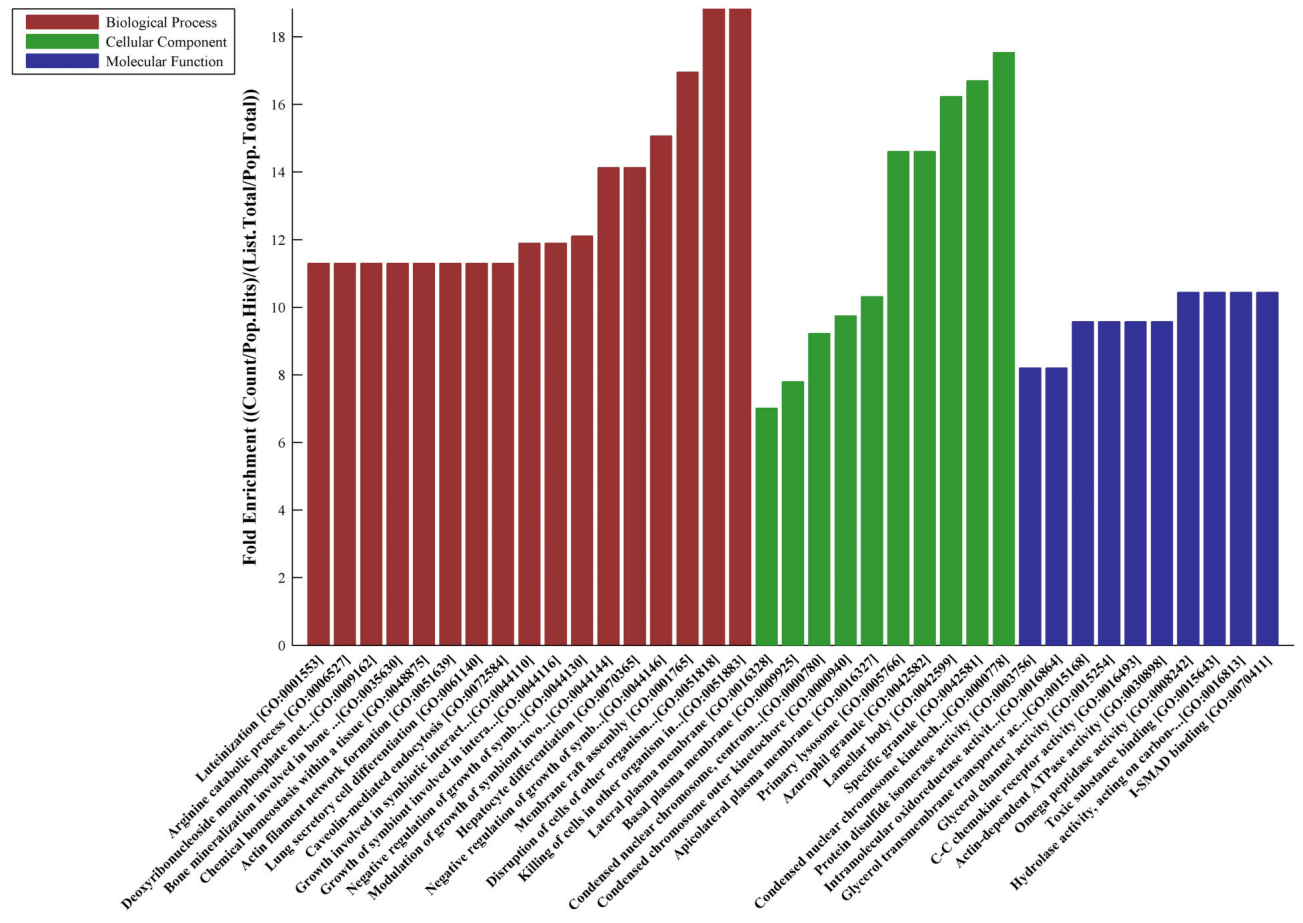
Analysis of the significant Gene Ontology terms (molecular function, cell component, and biological process analyses) for up-regulated genes.

**Figure 3.**
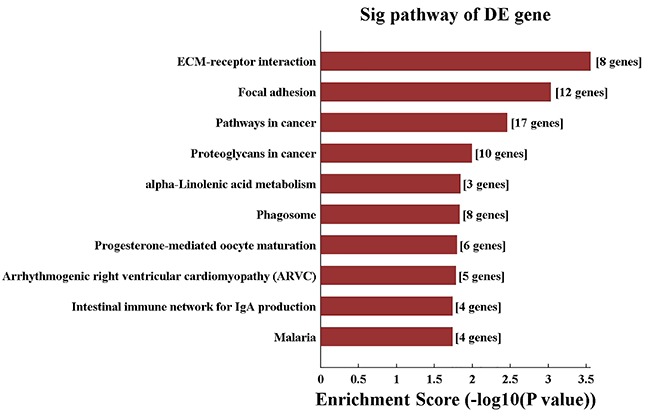
KEGG pathway analysis. The first ten pathways that exhibited significant differences (differentially expressed (DE) genes) between the 2 groups (P<0.005) are listed (up-regulated mRNAs). Seventeen genes known to be related to cancer were observed in pancreaticobiliary maljunction.

The results of the Gene Ontology (GO) analysis for up-regulated genes is shown in Figure 2. We also mapped the 876 DEGs to the KEGG (Kyoto Encyclopedia of Genes and Genomes) pathways to further identify target mRNAs and their common cellular processes. The pathways most common to the up-regulated genes are shown in Figure 3. Major pathways targeted by the up-regulated genes included those implicated in metabolism, receptor interaction, adhesion, and cancer pathway.

### qRT-PCR validation

**Figure 4.**
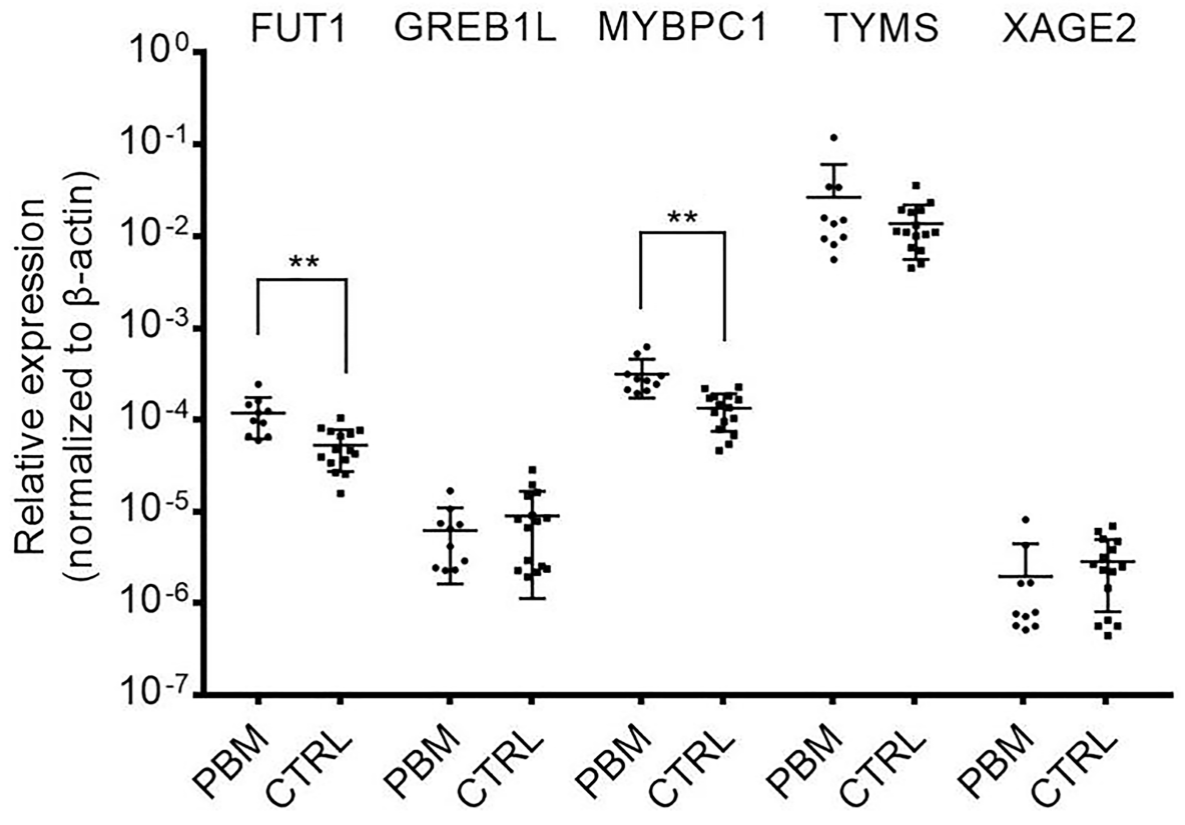
qRT-PCR validation of 5 selected differentially expressed genes in pancreaticobiliary maljunction (PBM). Each spot represents the gene expression value (corrected by β-actin housekeeping gene (ΔΔCt)) of an individual patient (n=10). Midline represents the mean. **P<0.001 (rank sum test). CTRL: control.

**Figure 5.**
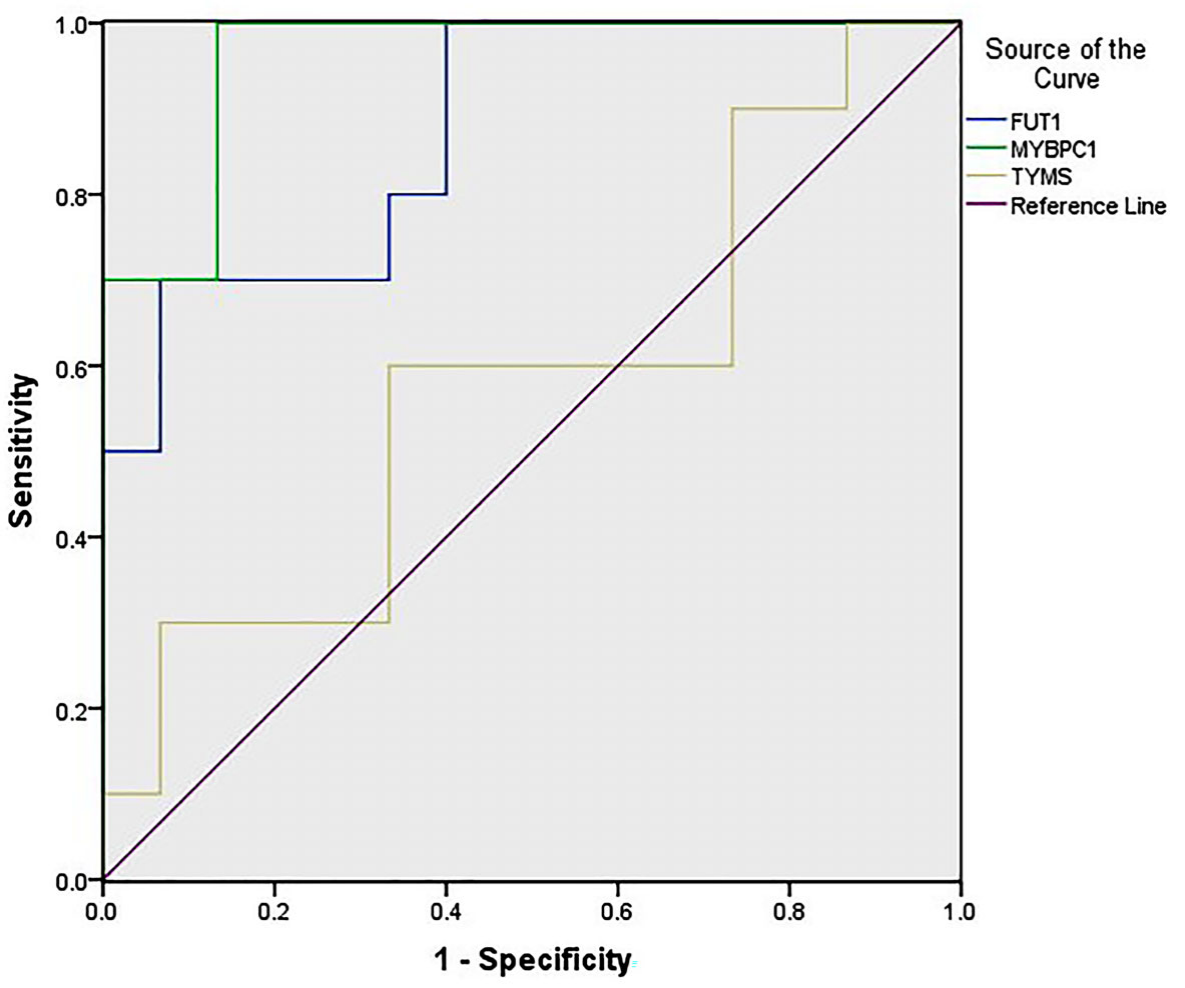
Receiver operating characteristic curve of *MYBPC1* and *FUT1* as biomarkers for pancreaticobiliary maljunction (PBM). Area under the curve = 0.960 and 0.873, respectively.

Up-regulation of *TYMS*, *MYBPC1*, *FUT1*, *XAGE2*, and *GREB1L*, as suggested by the microarray experiments, were verified by qRT-PCR. Up-regulation of *MYBPC1* and *FUT1* were successfully validated by qRT-PCR (P<0.01). qRT-PCR failed to validate the preliminary findings with *TYMS*, *XAGE2*, and *GREB1L* (Figure 4). The AUC under ROC curve was 0.960 and 0.873 for *MYBPC1* and *FUT1*, respectively (Figure 5).

## Discussion

Previous studies using a genome-wide approach identified altered gene expressions in the biliary tract of children with choledochal cyst/PBM. For example, Kaneko et al. examined genome-wide expression in gallbladder epithelia obtained from 6 children with PBM versus 4 pediatric controls using microarray analysis followed by RT-PCR verification (11). They found several dysregulated genes that may contribute to the pathophysiology of PBM. Wong et al. (12) conducted exome-sequencing in 31 pedigrees of congenital bile duct dilatation of the bile-duct cases and found association of six genes carrying damaging *de novo* variants with human developmental disorders involving epithelial and four linked with cholangio- and hepatocellular carcinomas.

In the present study, a group of 13 up-regulated genes with varying functional importance in the PBM group was identified. The top 5 on this list (*TYMS, MYBPC1*, *FUT1*, *XAGE2*, and *GREB1L*) were examined using RT-PCR. The RT-PCR experiments confirmed the up-regulation of *MYBPC1* and *FUT1*, but not *TYMS*, *XAGE2*, and *GREB1L*.

*FUT1* has been shown to be over-expressed in both human and rat colon cancers (13,14). A previous study by Zhang et al. (15) reported that suppressing the expression of *FUT1/4* by RNAi technology reduces the synthesis of LeY and inhibits cancer growth. The current study showed up-regulation of *FUT1* in PBM patients. Whether such an increase reflects the propensity of carcinogenesis requires further study.

*TYMS* is located on chromosome 18p11.32, and is composed of six introns with sizes ranging from 507 to 6271 bp and seven exons with sizes ranging from 72 to 250 bp (16–18). *TYMS* encodes for thymidylate synthase (TS), an enzyme indispensable for DNA synthesis and repair (19). TS level is under strict control and can be modified by genetic variations in the *TYMS* gene. Decreased TS activity decreases folate level and DNA repair capability, and consequently promotes carcinogenesis (20,21). Burdelski et al. reported that high *TYMS* expression is significantly associated with unfavorable tumor phenotypes, rapid tumor cell proliferation, and early recurrence of prostate cancer (22). In the present study, higher *TYMS* expression in PBM patients was found with microarray, but not confirmed with RT-PCR. Such a discrepancy may be due to the relatively small sample size, and remains to be verified in future studies.

*MYBPC1* encodes a member of the myosin binding protein-C family. Geist and Kontrogianni-Konstantopoulos (23) reported that *MYBPC1* and its isoforms interact with thick and thin filaments to regulate the cycling of actomyosin cross bridges. A previous study from this research group found higher expression of phosphorylated myosin regulatory light chain in the common bile duct in pediatric PBM (24), suggesting that *MYBPC1* up-regulation is a consequence of hypertrophy and fibrosis in common bile duct of PBM. *MYBPC1* up-regulation identified in PBM patients in the current study suggests that *MYBPC1* up-regulation could be used to predict pediatric PBM.

The current study has several limitations. First, the sample size is relatively small. Second, only five DEGs identified with microarray experiments were validated with RT-PCR. Third, only the top 5 up-regulated genes were validated by RT-PCR. Future studies with larger sample sizes and in-depth verification of more candidate genes using more definitive methods such as western blot are needed. Also, the relationship between genetic profiles and damage to the bile duct epithelium and eventual carcinogenesis requires further investigation.

In conclusion, *FUT1* and *MYBPC1* are up-regulated in children with PBM, suggesting myosin dysfunction. More detailed genetic profiling and functional analysis are warranted.
